# Association Between Serum Ferritin and Disease Severity in COVID-19 Patients in Bangladesh: A Cross-Sectional Study

**DOI:** 10.7759/cureus.109466

**Published:** 2026-05-22

**Authors:** Nafiza Afroz, Shahadat H Polash, Manas Mazumder, Jannatun Naim, Md Abdul Hamed Jasham, AKM Akhtaruzzaman

**Affiliations:** 1 Anesthesia, Intensive Care and Pain Medicine, National Institute of Burn and Plastic Surgery, Dhaka, BGD; 2 Critical Care Medicine, Dhaka Medical College Hospital, Dhaka, BGD; 3 Analgesia and Intensive Care Medicine, Bangladesh Medical University, Dhaka, BGD; 4 Critical Care Medicine, Directorate General of Health Services, Dhaka, BGD; 5 Critical Care Medicine, Continental Hospitals, Dhaka, BGD; 6 Anesthesia, Analgesia and Intensive Care Medicine, Bangladesh Medical University, Dhaka, BGD

**Keywords:** bangladesh, biomarker, covid-19, disease severity, roc analysis, serum ferritin

## Abstract

Background

Coronavirus disease 2019 (COVID-19) has a wide spectrum of severity, and early identification of high-risk patients remains a major clinical challenge, particularly in resource-limited settings. Serum ferritin, an acute-phase reactant, has been proposed as a potential biomarker of disease severity. This study aimed to evaluate the association between serum ferritin levels and COVID-19 disease severity and to assess its diagnostic performance as a biomarker for identifying severe-to-critical cases among COVID-19 patients in Bangladesh.

Methods

This hospital-based cross-sectional study was conducted between May and December 2021 at Bangabandhu Sheikh Mujib Medical University. A total of 46 reverse transcription polymerase chain reaction (RT-PCR)-confirmed adult COVID-19 patients were included via consecutive sampling, with equal distribution between the mild-to-moderate and severe-to-critical groups. Clinical data and laboratory parameters, including serum ferritin, were recorded at admission. Statistical analysis was performed via SPSS version 26. Group comparisons, logistic regression, and receiver operating characteristic (ROC) curve analyses were conducted.

Results

The mean age of the participants was 53.35 ± 15.9 years, and 23 (50%) participants were in each severity group. Serum ferritin remained significantly associated with disease severity in both the unadjusted (OR = 1.3, 95% CI: 1.03-1.33, p = 0.015) and age-adjusted (OR = 1.1, 95% CI: 1.01-1.23, p = 0.018) models. Neutrophilia, lymphopenia, and elevated D-dimer and C-reactive protein (CRP) levels were also significantly associated with severe disease (p < 0.001). ROC analysis revealed an area under the curve (AUC) of 0.76, with a cutoff value of 752 ng/mL, yielding 69.6% sensitivity and 73.9% specificity (p = 0.003).

Conclusion

Serum ferritin is significantly associated with COVID-19 severity and may be a useful, low-cost biomarker for identifying severe disease. It may serve as a potential indicator for clinical assessment. However, further large-scale multicenter studies are needed to validate its prognostic utility.

## Introduction

Coronavirus disease 2019 (COVID-19), caused by severe acute respiratory syndrome coronavirus 2 (SARS-CoV-2), was first reported in Wuhan, China, in December 2019 and subsequently spread across the globe, evolving into a major public health emergency. According to the World Health Organization (WHO), more than 700 million confirmed cases and over seven million deaths have been documented worldwide [1]. The clinical presentation of COVID-19 ranges widely from asymptomatic infection and mild symptoms to severe pneumonia, acute respiratory distress syndrome (ARDS), multiorgan failure, and death [2]. Among hospitalized patients, disease severity differs considerably, making early identification of high-risk individuals a significant clinical challenge [3].

Severe COVID-19 is primarily associated with an abnormal and exaggerated immune response, commonly described as a cytokine storm, characterized by an excessive release of proinflammatory cytokines. A meta-analysis demonstrated significantly elevated levels of interleukin-6 (IL-6), interleukin-8 (IL-8), interleukin-10 (IL-10), and tumor necrosis factor-alpha (TNF-α) in patients with severe disease, along with decreased CD4+ and CD8+ T-cell counts [4]. This hyperinflammatory state contributes to endothelial damage, coagulation disturbances, and organ dysfunction, highlighting the importance of inflammatory biomarkers in assessing disease severity and guiding clinical management [5].

Serum ferritin, an intracellular iron storage protein, also functions as a positive acute-phase reactant and increases markedly during systemic inflammation [6]. While it reflects iron stores under normal conditions, inflammatory cytokines such as IL-6 and interleukin-1 beta (IL-1β) stimulate ferritin production during infection, reducing its specificity for iron status [7]. In COVID-19, hyperferritinemia is considered both a marker and a mediator of immune dysregulation and has been linked to the concept of hyperferritinemic syndromes, where ferritin may play a pathogenic role [7].

Increasing evidence supports the role of serum ferritin as a biomarker of COVID-19 severity. A large meta-analysis by Kaushal et al. reported significantly higher ferritin levels in severe-critical patients than in mild patients (standardized mean difference (SMD) 0.882; 95% CI 0.738-1.026), with even greater elevations among nonsurvivors (SMD 0.992) and intensive care unit (ICU) patients (SMD 0.674) [8]. Similarly, Kurian et al. reported higher median ferritin levels in hospitalized patients with moderate-severe disease [9]. Receiver operating characteristic (ROC)-based studies further demonstrated its predictive value; Bozkurt et al. identified a cutoff of ≥264.5 ng/mL with 73.9% sensitivity and 94.2% specificity [10], whereas another study reported a cutoff of 608.9 μg/L with moderate accuracy [11]. Although variability exists, these findings highlight ferritin’s potential utility in identifying patients with greater disease severity.

Bangladesh, a lower-middle-income country, faced significant challenges during the COVID-19 pandemic. By November 2022, over two million cases and 29,431 deaths had been reported [12]. Limited healthcare resources, including only 733 ICU beds nationwide at the onset of the pandemic, constrained the management of severe cases [13]. Increased mortality and ICU utilization during subsequent waves further emphasized the burden on the healthcare system [14]. In such settings, cost-effective and widely available biomarkers such as serum ferritin are particularly valuable for early triage and resource allocation.

In low-resource environments, advanced diagnostic tools are often unavailable, and simple, accessible biomarkers are essential. The COVID-19 Low- and Middle-Income Countries (COVID-LMIC) Task Force recommends pragmatic triage strategies using low-cost tools [15]. The use of serum ferritin fits this need because of its availability and affordability. However, its performance may vary across populations, necessitating local evidence. Despite growing global research, data from Bangladesh remain limited. Therefore, the primary objective of this study was to evaluate the association between serum ferritin and COVID-19 disease severity, whereas the secondary objective was to assess the diagnostic performance of serum ferritin using ROC curve analysis.

## Materials and methods

Study design and setting

This was a hospital-based cross-sectional observational study carried out in dedicated COVID-19 units, including both the COVID ward and intensive care unit, of Bangabandhu Sheikh Mujib Medical University (BSMMU), Dhaka. The study was conducted over a one-year period from May to December 2021.

Study population

The study population comprised adult patients aged 18 years or older who were admitted with laboratory-confirmed COVID-19 infection. Diagnosis was established via reverse transcription polymerase chain reaction (RT-PCR) testing of nasopharyngeal or oropharyngeal swab samples. Patients were classified into two categories on the basis of national clinical guidelines: mild-moderate disease and severe-critical disease.

Inclusion and exclusion criteria

All RT-PCR-confirmed COVID-19 patients admitted to the BSMMU during the study period were considered eligible for inclusion. Patients with severe anemia, pregnancy, active malignancy, inability to cooperate, or who declined to provide informed consent were excluded from the study.

Sampling technique and sample size

The required sample size was determined by comparing the mean serum ferritin levels between mild-to-moderate and severe-critical COVID-19 patients on the basis of previously published data [16]. In that study, the mean ferritin level was reported to be 1074.53 ng/mL for the mild-moderate group and 3457.85 ng/mL for the severe-critical group, with corresponding standard deviations of 708.60 and 2817.60, respectively. For the calculation, a significance level of 5% (Zα = 1.96) and a statistical power of 80% (Zβ = 0.85) were assumed. The sample size was estimated via the standard formula for comparison of two independent means. Accordingly, the minimum required sample size was 23 patients in each group, resulting in a total sample size of 46 participants.

\[ n = \frac{(Z_{\alpha} + Z_{\beta})^{2} (\sigma_{1}^{2} + \sigma_{2}^{2})}{(\mu_{1} - \mu_{2})^{2}} \]

Data collection procedures

Data were obtained via a predesigned structured case record form. All eligible RT-PCR-confirmed COVID-19 patients admitted during the study period who fulfilled the inclusion criteria were approached consecutively for enrollment until the required sample size was achieved. Patients were recruited from both the COVID ward and intensive care unit (ICU) to ensure representation across different severity categories and to minimize selection bias. Information regarding sociodemographic characteristics, including age, sex, and smoking status; clinical presentation; existing comorbid conditions; and vital parameters such as pulse rate, blood pressure, respiratory rate, and oxygen saturation was documented at the time of admission. Laboratory evaluations included complete blood count, serum ferritin, D-dimer, and C-reactive protein (CRP) levels. Serum ferritin and other laboratory parameters were measured within 24 hours of hospital admission as part of the initial clinical evaluation. Serum ferritin levels were measured via chemiluminescent microparticle immunoassay (CMIA) in the Department of Biochemistry and Molecular Biology at BSMMU.

Assessment of disease severity

Disease severity was categorized in accordance with national COVID-19 management guidelines. Patients with mild-to-moderate disease were defined as those presenting with features of pneumonia without evidence of severe respiratory compromise (SpO₂ >90% and respiratory rate <30 breaths per minute). In contrast, severe-critical cases included patients exhibiting respiratory distress (respiratory rate ≥30/minute), hypoxemia (SpO₂ <90%), a PaO₂/FiO₂ ratio <300 mmHg, or those requiring mechanical ventilation, experiencing shock, or developing organ failure necessitating intensive care unit (ICU) support [17].

Statistical analysis

Statistical analyses were performed via IBM SPSS Statistics for Windows, Version 26 (Released 2018; IBM Corp., Armonk, New York). The distribution of continuous variables was assessed via the Shapiro-Wilk test, and the data are presented as means ± standard deviations. Differences between groups were evaluated via the independent samples t-test for continuous variables and the chi-square test for categorical variables. Binary logistic regression analysis was carried out to determine factors associated with disease severity, with serum ferritin included as a continuous predictor. To account for potential confounding, age-adjusted logistic regression analysis was additionally performed for serum ferritin and disease severity. ROC curve analysis was applied to assess the diagnostic performance of serum ferritin, including estimation of the area under the curve (AUC), sensitivity, specificity, and optimal cutoff points. There were no significant missing data for the primary study variables; therefore, complete-case analysis was performed. A p-value of less than 0.05 was considered statistically significant.

Ethical considerations

Prior to initiation of the study, ethical clearance was obtained from the Institutional Review Board (IRB) of BSMMU (approval no: BSMMU/2021/3961). Written informed consent was obtained from all participants or their legal attendants. Strict measures were taken to ensure the confidentiality and anonymity of the participants, and the study was conducted in compliance with the principles outlined in the Declaration of Helsinki.

## Results

The overall mean age of the participants was 53.35 ± 15.9 years. Patients in the severe-critical group were relatively older than those in the mild-moderate group (56.4 ± 15.1 vs 50.3 ± 16.5 years), although the difference was not statistically significant (p = 0.199). The proportion of participants aged ≥60 years was identical in both groups. A higher proportion of male patients was observed in the severe-critical group compared to the mild-to-moderate group, 14 (60.9%) vs 9 (39.1%), but this difference was not statistically significant (p = 0.119). In contrast, smoking status showed a statistically significant association with disease severity, with a greater proportion of smokers in the severe-critical group than in the mild-moderate group, 9 (39.1%) vs 3 (13.0%), p = 0.045. Comorbid conditions, including diabetes mellitus, hypertension, chronic kidney disease, chronic obstructive pulmonary disease/asthma, and obesity, were similarly distributed between the groups, with no statistically significant differences observed. Multiple comorbidities were present in an equal proportion of participants in both groups, 9 (39.1%) vs 9 (39.1%). Overall, the baseline characteristics were comparable between the two severity groups, except for smoking status (Table 1). 

**Table 1:** Baseline sociodemographic and clinical characteristics of the study population (n = 46) SD: standard deviation, CKD: chronic kidney disease, COPD: chronic obstructive pulmonary disease.

Variable	Category	Mild-Moderate (n = 23)	Severe-Critical (n = 23)	p value
Age (years)	Mean ± SD	50.3 ± 16.5	56.4 ± 15.1	0.199
Age group	<60 years	15 (65.2)	15 (65.2)	0.119
	≥60 years	8 (34.8)	8 (34.8)	
Sex	Male	9 (39.1)	14 (60.9)	0.119
	Female	14 (60.9)	9 (39.1)	
Smoking	Yes	3 (13.0)	9 (39.1)	0.045
	No	20 (87.0)	14 (60.9)	
Diabetes mellitus	Yes	11 (47.8)	12 (52.2)	0.500
	No	12 (52.2)	11 (47.8)	
Hypertension	Yes	11 (47.8)	10 (43.5)	0.500
	No	12 (52.2)	13 (56.5)	
CKD	Yes	1 (4.3)	3 (13.0)	0.304
	No	22 (95.7)	20 (87.0)	
COPD/Asthma	Yes	4 (17.4)	4 (17.4)	0.650
	No	19 (82.6)	19 (82.6)	
Obesity	Yes	1 (4.3)	0 (0.0)	0.650
	No	22 (95.7)	23 (100)	
Multiple comorbidity	Yes	9 (39.1)	9 (39.1)	0.618
	No	14 (60.9)	14 (60.9)	

The occurrence of cough was comparable between the mild-moderate and severe-critical groups (56.5% vs 60.9%, p = 0.500). Fever, however, was significantly more common among patients in the mild-moderate group, 18 (78.3%), than among those in the severe-critical group, 5 (21.7%) (p < 0.001). In contrast, shortness of breath was observed more commonly in severe-critical patients, 9 (39.1%), than in mild-moderate patients, 2 (8.7%) (p = 0.018). Similarly, respiratory distress was considerably more prevalent in the severe-critical group, 15 (65.2%), than in the mild-moderate group, 1 (4.3%), which was a statistically significant difference (p < 0.001). Other clinical features, including sore throat and malaise, did not differ significantly between the two groups (Table 2). 

**Table 2:** Clinical characteristics of the study population on admission

Variable	Category	Mild-Moderate (n = 23)	Severe-Critical (n = 23)	p value
Cough	Yes	13 (56.5)	14 (60.9)	0.500
	No	10 (43.5)	9 (39.1)	
Fever	Yes	18 (78.3)	5 (21.7)	<0.001
	No	5 (21.7)	18 (78.3)	
Sore throat	Yes	10 (43.5)	5 (21.7)	0.104
	No	13 (56.5)	18 (78.3)	
Malaise	Yes	10 (43.5)	5 (21.7)	0.104
	No	13 (56.5)	18 (78.3)	
Shortness of breath	Yes	2 (8.7)	9 (39.1)	0.018
	No	21 (91.3)	14 (60.9)	
Respiratory distress	Yes	1 (4.3)	15 (65.2)	<0.001
	No	22 (95.7)	8 (34.8)	

A comparison of the vital parameters between the two groups is presented in Table 3. The mean pulse rate was slightly greater in the severe-critical group (86.4 ± 20.14 bpm) than in the mild-moderate group (81.48 ± 10.25 bpm), although this difference was not statistically significant (p = 0.298). Similarly, the systolic and diastolic blood pressure values were greater among severe-critical patients, but these differences were not statistically significant (p = 0.329 and p = 0.477, respectively). The oxygen saturation levels were comparable between the groups (91.74 ± 7.48 vs 87.11 ± 2.21, p = 0.554). Overall, no significant differences were observed in baseline vital signs between the severity groups.

**Table 3:** Clinical signs of the study population on admission bpm: beats per minute, BP: blood pressure, SpO₂: peripheral oxygen saturation; SD: standard deviation.

Variable	Mild-Moderate (n = 23), Mean ± SD	Severe-Critical (n = 23), Mean ± SD	p value
Pulse rate (bpm)	81.48 ± 10.25	86.4 ± 20.14	0.298
Systolic BP (mmHg)	128.69 ± 10.57	136.48 ± 23.00	0.329
Diastolic BP (mmHg)	80.00 ± 7.97	84.17 ± 26.76	0.477
SpO₂ (%)	91.74 ± 7.48	87.11 ± 2.21	0.554

Significant differences in laboratory parameters were observed between patients with mild-moderate disease and those with severe-critical disease (Table 4). The neutrophil percentage was notably greater in the severe-critical group (82.00 ± 9.75%) than in the mild-moderate group (66.91 ± 16.26%), and this difference was statistically significant (p<0.001). In contrast, the lymphocyte percentage was significantly lower among severe-critical patients (10.83 ± 7.16% vs 26.56 ± 16.49%, p<0.001). The serum ferritin level was substantially greater in the severe-critical group (1833.35 ± 730.29 ng/mL) than in the mild-moderate group (513.45 ± 216.90 ng/mL), indicating a statistically significant difference (p=0.007). Similarly, D-dimer concentrations were significantly elevated in severe-critical patients (3.51 ± 3.26 mg/L) compared with mild-moderate patients (0.96 ± 0.88 mg/L) (p<0.001). CRP levels also significantly increased in severe cases (p=0.03). However, no statistically significant differences were found in the total white blood cell count or platelet count between the two groups.

**Table 4:** Laboratory findings of the study population WBC: white blood cell, CRP: C-reactive protein, SD: standard deviation.

Variable	Mild-Moderate (n = 23), Mean ± SD	Severe-Critical (n = 23), Mean ± SD	p value
WBC (per cmm)	7364.04 ± 5521.46	8600.16 ± 4593.35	0.413
Neutrophil (%)	66.91 ± 16.26	82.00 ± 9.75	<0.001
Lymphocyte (%)	26.56 ± 16.49	10.83 ± 7.16	<0.001
Serum ferritin (ng/mL)	513.45 ± 216.90	1833.35 ± 730.29	0.007
Platelet count	270791 ± 131730	227139 ± 114267	0.246
D-dimer (mg/L)	0.96 ± 0.88	3.51 ± 3.26	<0.001
CRP (mg/L)	110.5 ± 50.33	145.78 ± 57.05	0.03

Binary logistic regression analysis was performed to evaluate factors associated with disease severity (Table 5). Serum ferritin was identified as a significant factor associated with severe COVID-19 in both the unadjusted model (OR = 1.30, 95% CI: 1.03-1.33, p = 0.015) and the age-adjusted model (OR = 1.10, 95% CI: 1.01-1.23, p = 0.018), indicating that each 1 ng/mL increase in serum ferritin was associated with a greater likelihood of severe disease. Age was not independently associated with disease severity (p = 0.181).

**Table 5:** Logistic regression analysis for factors associated with severity OR: odds ratio, CI: confidence interval.

Variable	OR	95% CI	p value
Model 1: Serum ferritin	1.3	1.03-1.33	0.015
Model 2: Serum ferritin (age-adjusted)	1.1	1.01-1.23	0.018
Age	0.96	0.923-1.013	0.181

**Figure 1:**
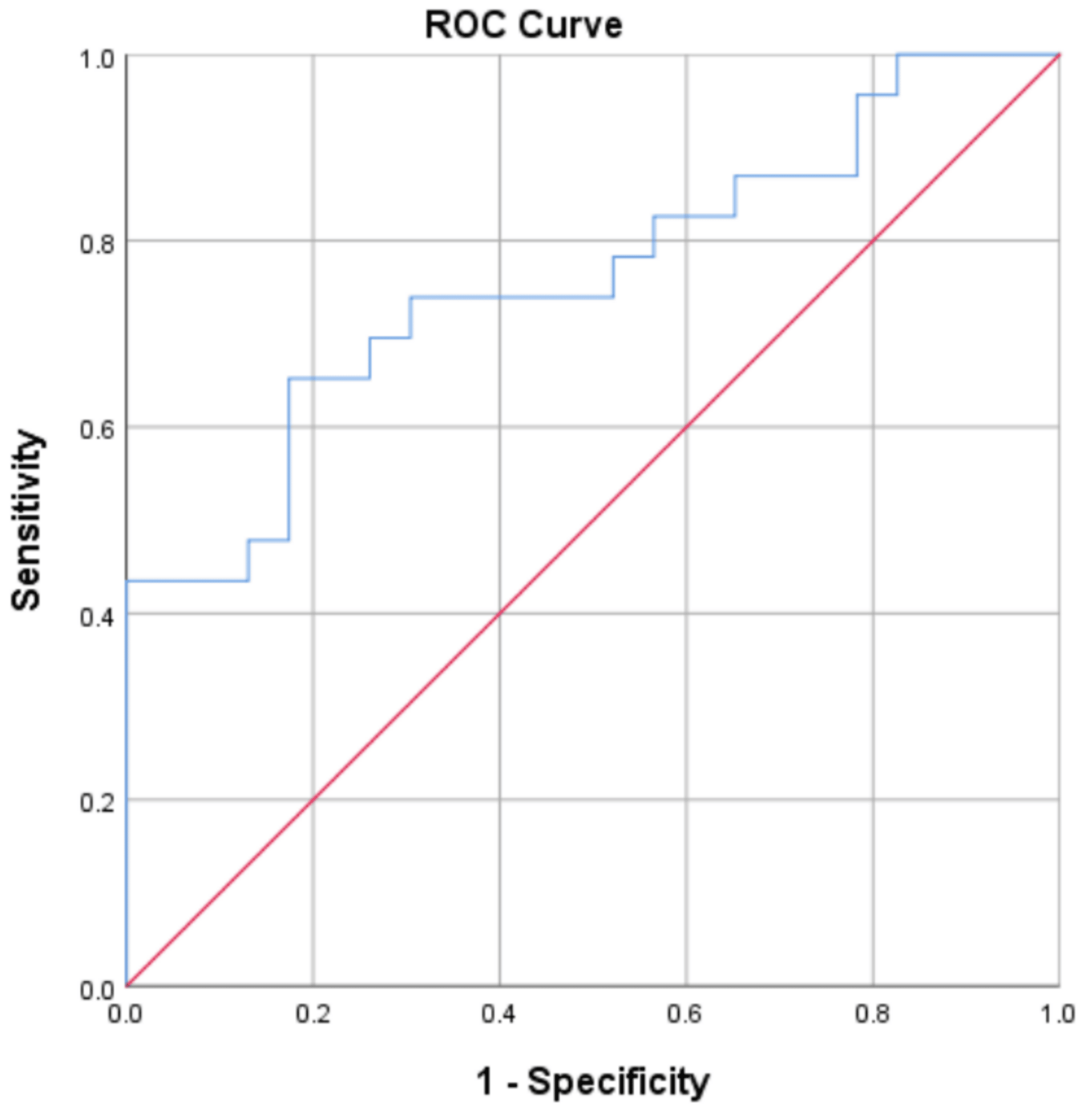
Receiver operating characteristic (ROC) curve showing the performance of ferritin in identifying the severity of COVID-19

The ability of the serum ferritin level to predict disease severity is summarized in Table 6 and Figure 1. ROC curve analysis revealed an AUC of 0.76, reflecting moderate diagnostic performance. An optimal cut-off value of 752 ng/mL was determined, yielding a sensitivity of 69.6% and a specificity of 73.9% (p = 0.003). These results indicate that serum ferritin has a moderate level of accuracy in identifying patients with severe COVID-19.

**Table 6:** Diagnostic performance of serum ferritin AUC: area under the curve, CI: confidence interval.

Parameter	Value
AUC	0.76
Cutoff value (ng/mL)	752
Sensitivity	69.6%
Specificity	73.9%
p value	0.003
95% CI of AUC	(0.65-0.85)

## Discussion

In the present study, we aimed to evaluate the association between serum ferritin levels and disease severity among COVID-19 patients. Our findings demonstrated that serum ferritin levels were significantly elevated in severe-critical COVID-19 cases and were significantly associated with disease severity. In addition, ROC analysis revealed moderate diagnostic accuracy of serum ferritin in identifying severe-critical disease.

One of the key findings of this study was significantly higher serum ferritin levels in severe-critical patients than in mild-moderate patients, which is consistent with the findings of previous studies and meta-analyses reporting a robust positive association between hyperferritinemia and COVID-19 severity. A comprehensive meta-analysis by Kaushal et al., encompassing 39 studies, demonstrated that severe-to-critical patients had significantly higher ferritin levels than patients with mild-to-moderate disease [8]. An observational study by Kurian et al. among 870 hospitalized COVID-19 patients similarly reported that median ferritin levels were substantially higher in patients with moderate-to-severe disease than in those with mild disease [9]. An earlier cross-sectional study from Israel by Dahan et al. likewise noted that severe patients had considerably higher ferritin concentrations than nonsevere patients [16]. These concordant observations across geographically diverse populations suggest a biologically plausible mechanism: during SARS-CoV-2 infection, excessive release of proinflammatory cytokines such as IL-6, IL-1β, and TNF-α, which are hallmarks of the cytokine storm, upregulates ferritin synthesis in hepatocytes and activated macrophages, rendering serum ferritin an acute-phase reactant that reflects the degree of systemic hyperinflammation [4]. Furthermore, Lin et al. demonstrated that serum ferritin was independently associated with disease severity, with multivariate logistic regression identifying ferritin as a significant risk factor for severe COVID-19 in their cohort [18]. The magnitude of ferritin elevation observed in our study may also reflect the immune dysregulation characteristic of Bangladeshi patients, potentially shaped by local patterns of comorbidity, nutritional status, and prior immunological exposures, underscoring the value of population-specific local evidence.

Another important observation in the present study was the significant neutrophilia and lymphopenia in severe-critical patients compared with mild-moderate patients, alongside markedly elevated D-dimer and CRP concentrations. These findings are consistent with prior evidence characterizing the immunological and hematological signatures of severe COVID-19. A large clustering study by Vafadar Lee et al. demonstrated that a hyperinflammatory immune pattern defined by elevated CRP, neutrophilia, and lymphopenia was independently associated with a severe disease course and worse clinical outcomes [19]. The lymphopenia observed in severe cases may reflect SARS-CoV-2-induced apoptosis and depletion of T lymphocytes driven by cytokine-mediated toxicity, which is consistent with findings from a meta-analysis showing significantly reduced CD4+ and CD8+ T-cell counts in severe patients compared with nonsevere patients [4]. In parallel with lymphopenia, an elevated neutrophil-to-lymphocyte ratio (NLR) has been identified across multiple studies as a readily calculable inflammatory index associated with disease severity [20]. The significant increase in D-dimer in severe cases in our cohort aligns with the established pathophysiology of COVID-19-associated coagulopathy, whereby immune-mediated endothelial injury and cytokine storms activate the coagulation cascade, producing a hypercoagulable state [21]. A systematic review and meta-analysis of 100 studies by Varikasuvu et al. confirmed that higher D-dimer levels were independently associated with disease progression, with an AUC of 0.69 for identifying disease severity [22]. Elevated CRP, a well-established nonspecific acute-phase reactant, further corroborated the systemic inflammatory burden in severe cases, which is consistent with extensive prior literature documenting its role as both a severity indicator and a therapeutic monitoring parameter [19].

The current findings suggest that serum ferritin has moderate but clinically meaningful diagnostic and clinical relevance in relation to severe COVID-19. In the logistic regression analysis, serum ferritin was significantly associated with disease severity in both the unadjusted and age-adjusted models, indicating that the association was not substantially confounded by age. The ROC analysis yielded an AUC of 0.76, with a cutoff of 752 ng/mL, which produced a sensitivity of 69.6% and specificity of 73.9%. These findings are broadly concordant with those reported by Bozkurt et al., who reported a ferritin cutoff of ≥264.5 ng/mL to predict severe COVID-19 with a sensitivity of 73.9% and specificity of 94.2% in a Turkish cohort, and those of Kurian et al., who reported a cutoff of 287.4 ng/mL with a sensitivity of 73.3% and specificity of 69.7% [9,10]. The higher cutoff value in our cohort may reflect differences in patient population, disease wave, and locally prevailing SARS-CoV-2 variants at the time of data collection. A recent cross-sectional study among 200 ICU patients in India by Hulkoti et al. similarly confirmed that serum ferritin levels were significantly correlated with clinical outcomes, including the neutrophil-to-lymphocyte ratio and computed tomography (CT) severity score, supporting the value of ferritin as a prognostic indicator in resource-limited settings comparable to Bangladesh [23]. While ferritin alone has moderate discriminatory ability, which may limit its standalone use as a diagnostic test, its affordability, reproducibility, and routine availability in hospital laboratories in Bangladesh make it a pragmatically attractive first-line triage biomarker, particularly for identifying patients who may require early escalation of care in settings where advanced monitoring is constrained.

This study represents one of the limited investigations conducted in Bangladesh exploring the relationship between serum ferritin levels and COVID-19 disease severity via both regression and ROC analyses, thereby contributing important local evidence. The application of standardized laboratory methods and clearly defined severity criteria enhanced the internal validity of the study findings. However, certain limitations need to be acknowledged. The single-center nature of the study, along with the relatively small sample size (n = 46), may affect the generalizability of the results. Moreover, the cross-sectional design prevents the determination of causal relationships or temporal associations. The absence of longitudinal follow-up further limits the evaluation of clinical outcomes over time. Furthermore, additional inflammatory and treatment-related confounders were not included in the multivariable adjustment models, which may have influenced the observed associations. In particular, smoking status and coexisting inflammatory conditions may have acted as residual confounding factors influencing serum ferritin levels and disease severity. Although consecutive sampling was used to reduce selection bias, the possibility of sampling-related bias cannot be completely excluded.

## Conclusions

Serum ferritin was significantly associated with COVID-19 disease severity, with markedly higher levels observed among severe-critical patients. Serum ferritin also remained significantly associated with severe disease after age adjustment, whereas ROC analysis demonstrated moderate diagnostic performance in identifying severe-critical cases. These findings suggest that serum ferritin may serve as a useful, low-cost biomarker for early clinical risk stratification and assessment of disease severity, particularly in resource-limited settings. However, larger multicenter studies with longitudinal follow-up are needed to further validate its diagnostic and prognostic utility in COVID-19 patients.
